# Aurally impressed, yet not more stressed: On the relationship between audiovisual realism, social anxiety, and presence in a virtual social stress scenario

**DOI:** 10.1371/journal.pone.0345565

**Published:** 2026-03-23

**Authors:** Sarah Roßkopf, Andreas Mühlberger, Felix Stärz, Matthias Blau, Steven van de Par, Leon O. H. Kroczek

**Affiliations:** 1 Department of Psychology, Clinical Psychology and Psychotherapy, University of Regensburg, Regensburg, Germany; 2 Institut für Hörtechnik und Audiologie, Jade Hochschule, Oldenburg, Germany; 3 Cluster of Excellence Hearing4All, Germany; 4 Acoustics Group, Carl von Ossietzky University, Oldenburg, Germany; Universidad Nacional de Tres de Febrero, ARGENTINA

## Abstract

Binaural auralizations can create spatial hearing impressions that closely resemble real sound sources, enhancing immersion and realism in virtual environments. Although social interactions often involve emotional responses such as stress (e.g., during a job interview), the interplay between emotion and binaural auralizations in virtual social interactions remains underexplored. Therefore, we investigated the effects of audiovisual realism in a virtual social stress scenario based on the Trier Social Stress Test. Acoustic realism was manipulated between subjects using head-tracked binaural auralizations and a diotic condition. For binaural auralizations, simulated binaural room impulse responses were based either on individual or generic head-related impulse responses. Stressfulness was also varied: a control group performed a task with reduced cognitive demand and social-evaluative threat by only “testing” a virtual job interview scenario and reading aloud preformulated answers. Social presence, stress responses (measured by salivary cortisol, heart rate, and self-reports), and gaze behavior were assessed in 78 participants. The virtual scenario reliably induced stress across all audio conditions compared to the control version. Binaural auralizations were rated as more externalized and realistic than diotic audio, but did not significantly influence social presence, stress responses, or gaze behavior. Social presence increased with higher social-evaluative threat and over time. Social anxiety was associated with greater social presence, altered gaze behavior (shorter latencies), and, to some extent, stronger stress responses. It also interacted with the auralization type in affecting social presence. Overall, enhancing acoustic realism with externalized auralizations did not affect stress or presence in the virtual scenario. Elevated stress levels also in the control condition may have masked potential audio effects, implicating the need for investigating binaural auralizations in less stress-related social contexts.

## Introduction

Virtual Reality (VR) is a technology used to simulate compelling three-dimensional scenes evoking the feeling of actually being there, known as presence. Although interactivity and multisensory stimulation is known to improve presence, typically visual attributes of the virtual scene are modulated to enhance presence, such as the display’s resolution [1,2]. Recently, the implementation of binaural auralizations, and therefore enhancements of the virtual acoustic scene, has been used to improve audio-visual plausibility and presence [3]. However, the impact of binaural auralizations on presence and emotional responses in an interactive virtual social scenario which is associated with stress, is still unknown.

Virtual acoustics aim to simulate how an auditory event would sound in a specific environment. Often, the virtual sound sources are reproduced via headphones [4]. An important attribute to describe realistic binaural hearing is the externalization of the perceived auditory events. Externalization refers to the phenomenon where a sound source is perceived as coming from the surrounding environment, rather than from inside the head, as is typically the case with headphone playback of stereo or mono sound [5]. Therefore, both surrounding and vividness of VR are increased by implementing spatialized sound, positively affecting immersion and realism [6]. Typically, data on the user’s head orientation is retrieved from the head-mounted display (HMD) and combined with the corresponding binaural room impulse response (BRIR), allowing the human sensory system to perceive a stable sound source which is located in the virtual room. Despite the improvement in the realism of VR due to the use of spatialized sound, it remains unclear whether and how these improvements modulate user experience in VR. On the one hand, presence was found to be enhanced by spatial sound [7–9]. On the other hand, no consistent effects were found on social presence, a sub-aspect of presence which is especially relevant for VR applications in the context of, e.g., virtual social interactions. Social presence, the sense of being with another, as defined by Oh et al., is also influenced by the immersive qualities of VR [10]. In their review, the positive effects of higher audio fidelity on social presence were summarized [10]. However, only non-immersive environments presented on 2D screens or audio-only VR were investigated. Regarding immersive environments, in a recent study, binaural auralizations resulted in higher social presence ratings in direct comparison to less immersive sound in a virtual seminar room scenario [3]. Also, a positive effect of including individual head-related impulse responses was found, although this required a time-consuming measurement process. Contrarily, no benefits of binaural auralizations on social presence and communication behavior were found in a dyadic communication VR scenario [11]. Overall, implementing binaural auralizations can be expected to improve the quality of VR experience in terms of realism and presence, but the effects on social presence in virtual social interactions remain unclear.

Human social interactions are complex and challenging, often evoking emotional responses [12,13]. In VR, these affective experiences can be explored in a standardized manner, as even simply designed avatars can evoke substantial feelings of social presence [14,15]. Virtual social interactions are not only a helpful tool in communication and collaboration research, but can be used for basic research on stress and its neurophysiological basis [12,13]. The Trier social stress test (TSST) is the laboratory gold standard for investigating acute stress, especially concerning the neuroendocrinological domain [16]. Typically, an increase in salivary cortisol is used as an indicator of stress. Furthermore, physiological stress reactions are investigated using the TSST, e.g., by measuring heart rate [17], skin conductance [12], myoelectrical activity, or body temperature [18]. Finally, self-reports on affect and stress are typically collected, e.g., [13]. The complexity of human stress is reflected in differential response patterns, which are influenced not only by inter-individual traits but also by situational and contextual factors [13,17].

While conducting the TSST in VR offers several advantages in terms of logistical effort and standardization [16], not all studies found that the VR-TSST evoked a (neuroendocrinological) stress response. In a direct comparison study, it was further found that the VR-TSST evoked equal (or even higher) subjective stress, but decreased cortisol responses compared to the in-vivo version [13]. These findings were linked to the degree of immersion. More specifically, low levels of social presence in some VR-TSST studies were suggested to contribute to the differential effects [13]. The stress induction via TSST is mainly driven by social evaluative threat and uncontrollability [16,19]. In order to trigger this threat, subjects need to feel that others are present, and thus, a certain degree of social presence is required.

The impact of social evaluative threat on stress is also reflected in TSST studies, including patients with social anxiety disorders. These patients experience an intense fear of social situations involving potential judgment or embarrassment, resulting in daily-life impairments [20], and show stronger subjective stress reactions than healthy controls, but unaltered cortisol responses to the TSST [21]. Furthermore, socially anxious participants were found to experience higher levels of social presence [22]. In general, the effectiveness of VR applications, whether for the treatment of mental disorders [23] or for social skills training [24], increases with immersion [25].

The implementation of spatial audio, by using head-tracked binaural auralizations, may help to increase social presence within virtual scenes and therefore the impact of virtual social situations. If the sound of speech is perceived in the same location as a virtual agent, it may feel more like a naturalistic interaction compared to seeing a speaking agent but perceiving the produced sound inside the head. It is therefore to be investigated whether increased audiovisual realism affects social presence and, in turn, increases social stress reactions in a stressful virtual interaction. While the effectiveness of an intervention in VR seems to depend on (social) presence, it was also found that the immersivity of a virtual environment may be less relevant in demanding situations especially when anxiety is high [26]. The affective states, especially arousal and fear, are in a mutual relationship with presence [27]. Initial increases in fear positively affect presence, and enhanced presence in turn intensifies the fear reaction towards virtual phobic stimuli [28]. Therefore, the effect of binaural externalized auralizations in virtual social interactions is to be investigated under different stressful conditions. The potential positive effect of spatial sound may be higher in a social situation with comparable low social evaluative threat due to the interrelation of immersion, arousal, and presence. Furthermore, potential influences of the participants’ social anxiety trait should be considered. Since a relevant application of virtual social interactions is in the treatment of social anxiety disorders, differential effects of binaural auralizations on stress response and social presence are to be investigated.

To the best of our knowledge, no systematic evaluation of the influence of binaural auralizations on the induction of stress and social evaluative threat has been conducted. Therefore, this preregistered study (https://osf.io/7gy3p) aimed to investigate how binaural auralizations in a virtual social stress scenario (VST) affects presence and social presence as well as stress reactions (neuroendocrinological, physiological, and subjective). If the binaural auralizations are perceived as externalized, we refer to them as externalized auralizations. For that reason, we manipulated the social stress scenario (low vs. high-stress) and investigated low- versus high-socially anxious participants. A subsequent research question was whether individual acoustic measurements are necessary to simulate the externalized auralizations to maximize the effects. We derived the following hypotheses:

H1: Externalized auralizations increase social presence in virtual interactions compared to non-externalized ones.

H2: The VST evokes stronger stress responses in terms of higher increases of a) salivary cortisol, b) heart rate, and c) stress ratings from baseline to post-stress measurements (or during stress measurement for heart rate) when externalized auralizations are used.

H3: Concerning gaze behavior, we expect enhanced visual spatial attention when externalized auralizations are used, in the form of shorter latencies for the first fixation on virtual speakers.

H4: We hypothesize that the difference in levels of social presence between externalized and non-externalized auralizations is lower in the high-stress condition compared to the low-stress condition.

H5: In the high stress condition involving the externalized auralizations, social anxiety is a stronger predictor for social stress than in the high stress condition involving the non-externalized ones.

H6: Equivalency of individualized and generic externalized auralizations: Based on our previous findings [3,29,30], we expect that using individualized measurements for the externalized audio condition will not result in further improvements concerning social presence, stress reactions, or visual spatial attention.

## Methods

### Participants

Our sample (*N* = 78) consisted of 52 female and 26 male participants. No one identified as non-binary. Due to legal and hormonal reasons, only adults between 18 and 55 years were included. Our sample consisted mainly of young adults aged between 18 and 39 (*M* = 23.9, *SD* = 3.8). The sample size was based on a power analysis conducted with G*Power 3.1 [31], indicating N = 42 (14 non-externalized vs. 14 externalized–individual and 14 externalized-generic participants) to be sufficient to detect an effect size of d = 1.10 with alpha set at.05 and 1 – beta = .95 for a one-sided paired sample t-test (externalized vs. non-externalized). In a previous study [3], we found effect sizes of d > 1.10 for the comparison of externalized auralizations with the anchor control condition concerning social presence (primary outcome variable). We increased the sample size to N = 78 to have at least 13 participants per *Stress* x *Audio* group. The majority of participants were students (*n* = 71). Table 1 shows demographic, psychological, and further relevant characteristics of the experimental groups. We examined whether the experimental groups differed prior to the manipulations. As shown in Table 1, no differences emerged regarding outcome variables. Groups were also comparable in demographic and clinical characteristics, except for negative affect. Participants were recruited via the university’s participant management system and social media. All reported unimpaired hearing, normal or corrected vision, and at least five years of German-speaking experience (two were non-native speakers). No participant met criteria for a current affective episode, generalized anxiety disorder, or acute suicidal tendencies as confirmed with the Mini International Neuropsychiatric Interview (M.I.N.I., [32]). None reported current psychotherapy, psychotropic medication, cardiovascular or neurological conditions, tinnitus, or acute respiratory, sinus, or ear infections.

**Table 1:** Participants’ characteristics per experimental conditions.

	high-stress (*n* = 39)				low-stress (*n* = 39)							
	non-externalized		externalized		non-externalized		externalized		Stress		Audio	
	(n = 13)		(n = 26)		(n = 12)		(n = 27)		t or χ^2^	p	t or χ^2^	p
*Sex, male, n (%)*	4	31	9	35	4	33	9	33	0	1	<0.01	1
*Women using hormonal contraception, n (%)*	5	38	6	23	5	42	9	33	0.36	.549	1	.317
*Women in luteal phase or irregular cycle, n (%)*	4	31	13	50	4	33	11	41	0.02	.882	0.16	.692
*Age, y, M (SD)*	24.1	3.2	23.8	3.6	24.1	2.8	23.8	4.8	0	1	−0.37	.710
*Depression Screening (BSI-D), M (SD)*	1.4	0.4	1.2	0.3	1.3	0.4	1.3	0.4	0.41	.682	−1.06	.296
*Social Anxiety (SPIN), M (SD)*	35.2	11.7	29.1	11.6	27.9	9.8	28.2	11.5	1.17	.244	−1.11	.274
*Positive Affect (PANAS), M (SD)*	3.1	0.7	2.8	0.5	2.8	0.7	2.9	0.6	−0.02	.985	−0.49	.625
*Negative Affect (PANAS), M (SD)*	1.5	0.4	1.5	0.4	1.2	0.2	1.3	0.2	2.78	.007	0.20	.840
Baseline												
*Salivary Cortisol Level in nmol/l, M (SD)l*	3.4	2.7	5.0	5.0	3.9	3.5	3.0	1.7	1.49	.143	0.36	.719
Stress Rating, M (SD)	41.3	26.9	38.3	23.9	27.5	17.6	41.2	24.6	0.49	.628	0.92	.360
Heart Rate, M (SD)	92.8	15.0	86.5	14.3	83.0	12.4	87.5	13.4	0.69	.490	−0.30	.763

Abbreviations: BSI-D, Brief-Symptom-inventory-Depression; SPIN, Social phobia Inventory; PANAS, Positive Affect Negative Affect Scale; *χ*^*2-*^tests were conducted for categorical data, and t-tests were conducted for continuous variables.

Furthermore, measures were taken to reduce disruptive influences on salivary cortisol levels. Self-reporting pregnancy, lactation, or intake of medication containing glucocorticoids such as cortisol were defined as exclusion criteria, as well as regular smoking (more than 5 cigarettes per day). To control for menstrual cycle effects on cortisol, female participants were tested during the luteal phase (2–3 weeks after self-reported cycle onset). Females using hormonal contraception (n = 23) or self-reporting no regular cycles were tested independently of the current cycle. To control for circadian effects on cortisol, especially the cortisol awakening reaction [33], the experiments took place between 1 and 8 p.m. Participants were instructed to abstain from cannabis or any other psychotropic substances for three days, and from nicotine and alcohol for one day prior. Ninety minutes before testing, participants were instructed not to brush their teeth or eat a large meal. During the experiment, only water was allowed.

The study was realized in compliance with the Declaration of Helsinki and was approved by the ethics committee of the University of Regensburg (Ref-No.: 20-1804-101). All participants gave written informed consent. The study was conducted from December 2023 to July 2024. Participants received financial compensation, or psychology students, if preferred, course credits.

### Study design

The study employed a between-subjects design manipulating *Audio* (non-externalized, externalized-individual, externalized-generic) and *Stress* (low vs. high). The low-stress condition involved reduced social-evaluative threat and cognitive demand. Time was a within-subjects factor due to the repeated measures (stress responses, ratings). Primary outcomes included self-reported social presence via questionnaire (MPS), salivary cortisol increase, heart rate, and subjective stress. Gaze behavior was analyzed via first fixation latency, dwell time, and accuracy of first fixations on virtual agents. Secondary outcomes included in-VR social presence ratings, cortisol responder rates, adverse effects, and perceived audio quality. Social anxiety was analyzed as an individual difference factor.

### Materials

#### Low vs. high social stress manipulation.

To induce psychosocial stress in a controlled and standardized manner, we used an adaptation of the virtual reality version of the Trier Social Stress Test (VR-TSST [13,34]). To enhance the salience and potential impact of the audio condition, the VST included a higher proportion of committee speech. An introductory phase was added, during which virtual agents presented their roles and expertise. Instead of the standard VR-TSST math task, participants completed a question-and-answer (Q&A) round with 30 challenging job interview questions, each followed by 20 seconds for spontaneous responses. At the start of the VR, participants received condition-specific instructions. The high-stress group was told they would undergo a job interview for their “dream job” and should perform at their best. The low-stress group was informed they were testing a VR training scenario and should simply read prewritten answers aloud.

#### Virtual reality set-up.

**Fig 1:**
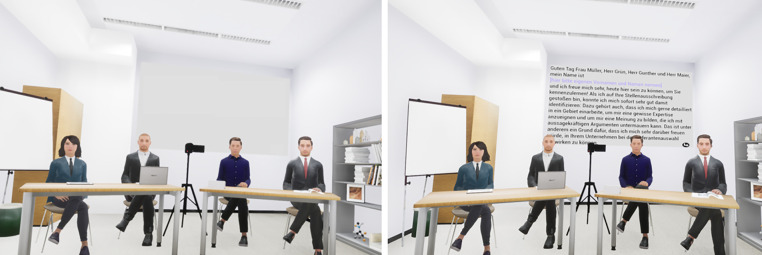
Virtual Stress Scenario. Left: high-stress; right: low-stress condition.

The virtual committee consisted of three males and one female who were formally dressed (suits, costumes, see Fig 1). They were created using MetaHumans (*MetaHuman Creator, Unreal Engine, & Quixel Bridge; Epic Games*) and Blender (v2.79, Blender Foundation). Their expressions were emphatically neutral, and they gave no feedback throughout the interaction to trigger social evaluative threat. All verbal interactions were pre-recorded voiceovers triggered by the VR game engine (see section 2.3.3). Lip synchronization was realized using Audio2Face AI (NVIDIA Omniverse^TM^).

The VST took place in a small seminar room of the University of Regensburg. We created two photorealistic models of this seminar room with the Unreal Game Engine (v 4.27, Epic Inc.) and Blender (v 2.79). One room was used for the job interview, and was equipped with the committee behind tables with tablets, writing materials, a whiteboard, a camera, etc. The other room was the preparation room and was equipped with a table, chair, and a notebook on which further instructions were written. The visual virtual environment was presented via an HMD (Vive Pro Eye, HTC). An inaudible work station with passive cooling was used (Silentmaxx PC Kenko S-770i). The starting position of participants in the physical room was matched to the corresponding position in the virtual visual room model via an in-house-developed two-point calibration technique using custom-made mounts for the HTC motion controller [35].

#### Audio set-up.

Three different auralization types were used. Two of the three auralizations (individual and generic HRIRs) were simulated in such a way that they evoke highly spatialized, realistic, and externalized hearing impressions (“externalized auralizations”). The third auralization was a “diotic” rendering, which should evoke a non-externalized hearing impression since binaural cues were eliminated. The auralizations were generated based on BRIRs simulated with RAZR (v0.962b; [36]). The simulations incorporated the dimensions of the experimental room (6.8 m × 4.8 m × 3.3 m), source directivity of loudspeakers (Genelec 8030b, Genelec Oy), and frequency-dependent absorption coefficients averaged in octave bands per room wall. The simulated reverberation time (T_20_ = 0.8s) was fitted to the physically measured monaural impulse responses of the experimental room. For the individualized auralization, BRIRs were simulated based on individual HRIRs, whereas the generic condition, HRIRs from a head-and-torso simulator (KEMAR Type 45BB, GRAS Sound and Vibration A/S, Holte, Denmark) were used. All HRIRs were recorded using a measurement setup that replicated the system developed at Jade Hochschule Oldenburg, see [29] for details. The simulations covered 37 azimuthal orientations (−90° to +90° in 5° steps) and nine elevation angles (−30° to +30° in 7.5° steps), with a fixed ear height of 1.60 m. For the diotic auralizations, the left and right BRIRs of the generic condition were averaged. The auralizations were combined with individualized headphone equalization and real-time head tracking via the HMD.

Audio was presented through extra-aural headphones (AKG K1000, AKG Acoustics GmbH, Vienna, Austria) mounted on the HMD using custom 3D-printed holds [37], powered by a headphone amplifier (Lake People G103P, Lake People Electronic GmbH, Konstanz, Germany) and an external audio interface (RME Fireface UC, Audio AG, Haimhausen, Germany).

Dry recordings of four trained speakers were used to generate the auralizations. These recordings were loudness-normalized using the integrated loudness function from MATLAB’s Audio Toolbox™ (following EBU R 128) to minimize loudness differences between individual speakers and Hann-windowed (10 ms per flank) to prevent onset or offset artifacts. The total duration of the speech was 5 minutes and 19 seconds.

### Procedure

#### Audio measurements and externalization instruction.

The experimental procedure comprised two appointments. During the first, participants gave written informed consent and completed psychoacoustic measurements based on their assigned audio condition. Those in the externalized-individual group underwent the measurement of HRIRs, which took approximately 30 minutes (for further details, see [29]). All participants underwent a headphone impulse response measurement (about 5 min). They were then introduced to the concept of externalization to prepare them for the related ratings during the second appointment. They were shown the externalization rating scale (see S2 Table) ranging from “0: fully inside the head” to “100: fully outside”. The instructor explained that typical television or a loudspeaker sound corresponds to full externalization (100), while headphone audio is usually perceived non-externalized (0), with intermediate perceptions also possible. To familiarize participants with the scale, they rated three binaural auralizations and one non-spatialized audio sample. For this procedure, no head-tracking was used, and the auralizations, audio stimuli, and headphones (model HD 800, Sennheiser electronic GmbH & Co. KG, Wedemark, Germany) were different from those of the VST.

#### Main experiment.

**Fig 2:**
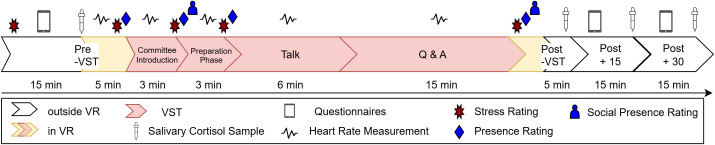
Experimental Procedure of the VST. Key measurement time points inside and outside VR.

*Pre-assessments and preparation* The second appointment started with general instructions, a check for exclusion criteria, and three sections of the neuropsychiatric interview. Afterwards, participants completed questionnaires (for further information on all used questionnaires see section Measurements – Self-Reports), first the demographic, followed by the BSI-17, the PANAS, and then the SPIN. Next, the first cortisol saliva sample was collected, the electrocardiogram (ECG) electrodes were attached, and psychophysiological recording started. The participants were then familiarized with the HMD and the controller before entering the experimental room, guided to the starting position by the experimenter and virtual footprints. Only then was the virtual replication of the room displayed. After eye tracking calibration, a 90-second ECG baseline was recorded while participants stood still and upright. Practice trials introduced the rating procedure, followed by the first ratings (Fig 2).

*VST* After the first ratings, VR scenes varied according to the experimental condition. In the low-stress group, participants were told they were testing a VR job interview training tool and instructed to read aloud predefined answers and a written talk. In the high-stress group, participants were asked to imagine applying for their dream job, to perform at their best, deliver a talk, and answer questions spontaneously. After confirming the instructions via button press, all participants were teleported to the virtual interview room (see Fig 1), where virtual agents introduced themselves (committee introduction) and instructed them to prepare a short talk. Participants were then teleported to a virtual preparation room with a desk and notebook, aligned with physical furniture. There, they completed further ratings and had three minutes to either prepare their talk (high-stress) or read the predefined version (low-stress), followed again by ratings (preparation phase). Participants then stood up, returned to the interview room, and began their talk upon instruction. After six minutes, a virtual agent ended the talk and initiated the Q&A with 30 challenging job interview questions (see S1 Table), adapted from an online source [38]. Each question began with a turn-taking sequence between the previous and current virtual speaker. Participants had 20 seconds to respond, spontaneously in the high-stress condition, or by reading predefined answers in the low-stress condition. The questioning agent ended each sequence with a brief, neutral remark (e.g., “Mhmm, thank you.”). After the final question and a closing statement by an agent, participants completed ratings on the experience of stress, VR and subjective audio quality features (see S2 Table). They then left the room and removed the HMD.

*Post-Assessments* Immediately after the VST, the second saliva sample was collected. Participants then completed further questionnaires via tablet: MPS, SSQ, qualitative questions on (auditory) VR experience, PANAS, SVF-78, hearing-related questions (e.g., musical experience, audio sensitivity), and the DAS-18. To assess peak cortisol response, two additional saliva samples were collected 15 and 30 minutes post-VST, following Dickerson & Kemeny [39]. Finally, the instructor checked participants’ current affective state and offered a referral to the university outpatient clinic if needed (no cases occurred).

### Measurements and preprocessing

#### Self-reports.

Subjective data was assessed using several questionnaires and analog rating scales implemented within the VR scene. Each of these scales included a rating item (see Table 2), with verbal anchors at both ends, 0 (“not at all”) and 100 (“very much”), and a slider that participants could adjust continuously using the HTC motion controller. Outside VR, all subjective data were collected using a tablet (Apple iPad Pro, 12.9-inch, 4th generation, model year 2020) and SoSci Survey (Version 3.1.06 [40]).

**Table 2:** Cortisol Responder Rate (in %) per experimental condition.

	High Stress (n = 39)	Low Stress (n = 39)	
**Externalized Auralizations (n = 53)**	42%	19%	30%
**Non-Externalized Auralizations (n = 25)**	42%	15%	28%
	41%	18%	

Stress was rated before VR (slider rating on tablet): “How stressed do you feel on a scale from 0: not stressed at all - to 100: maximally stressed?”, and during VR with the three stress items depending on the time point of measurement (see S2 Table). For each participant, the peak subjective stress level was defined as the highest self-reported stress following the onset of the VST. The German version of the MPS, the multimodal presence scale [41,42], was used for standardized (social) presence measurement. Also, the presence ratings within the VR scene (see S2 Table) were based on subitems of the MPS.

Further questionnaires were used. We used the SSQ, the simulator sickness questionnaire [43], to assess possible adverse effects of VR. The occurrence of psychopathological symptoms was assessed with the BSI-18, the brief symptom inventory [44]; the current affective state with the PANAS, the positive and negative affect schedule [45]; and social anxiety with the SPIN, the social phobia inventory [46]. The subscale of the Sensorik Inventar for hearing [47] was used as an indicator of audio sensitivity. To gain insights on stress-management, we assessed coping strategies with the SVF-78, the “Stress Verarbeitungsfragebogen” [48], and dysfunctional cognitions with the DAS-18, the dysfunctional attribute scale [49]. Fig 2 gives an overview of the measurements at several time points.

#### Heart rate.

To assess heart rate as a physiological indicator of social stress, we continuously recorded ECG data throughout the VST. Therefore, three self-adhesive electrodes (Ag/ AgCl, Ø = 40 mm; Diagramm Halbach GmbH & Co. KG, Schwerte, Germany) were attached to the participant, one on the sternum and one on each side of the lower costal arch. ECG data were collected using a portable wireless sensor (PLUX – Wireless Biosignals, S.A., Lisbon, Portugal). Data acquisition and storage were managed using the OpenSignals software (PLUX) and LabRecorder (Lab Streaming Layer, GitHub repository, 2014). The ECG recordings were analyzed offline using a custom MATLAB script (v R2022a, The MathWorks, Inc., Natick, MA, USA). Heart rate data were segmented into 30-second intervals, and the mean beats per minute (bpm) were computed for each segment and labeled with the respective experimental phase using markers sent by the VR engine. Further preprocessing was performed in the R statistical environment, Version 2024.04.2, [50]. The heart rate during the first 90 s of each segment was averaged and used for further analyses, as this corresponded to the length of the baseline measurement. Data from 10 participants had to be excluded due to technical errors, missing data, or markers.

#### Cortisol.

Salivary samples to determine cortisol levels as a neuroendocrinological indicator for social stress were taken at four time points (pre-VST, post-VST, post+15, post+30) using salivette collection tubes (Sarstedt AG & Co., Nümbrecht, Germany). After the experiment, the saliva samples were stored at −20°. They were analyzed in single determination (standard) at the laboratory of Prof. Dr. Clemens Kirschbaum in Dresden, which provided the following rationale: “After thawing, samples were centrifuged at 3,000 rpm for 5 min, which resulted in a clear supernatant of low viscosity. Salivary concentrations were measured using a commercially available chemiluminescence immunoassay with high sensitivity (Tecan - IBL International, Hamburg, Germany; catalogue number R62111). The intra- and interassay coefficients of variance were 2.2% and 2.9%. Three of the saliva samples were missing data (from a total of 312 samples). Participants (n = 2) with missing cortisol data (at baseline or peak) were excluded from analyses concerning salivary cortisol levels.”

Salivary cortisol levels were log-transformed (base 10) to normalize data. For statistical analyses, self-reported gender, age, and hormonal contraception (hc, 3 factors: male, female-no-hc, female-hc) of participants were included as covariates (Bärtl et al., 2024). Furthermore, for each participant, the peak cortisol level of salivary samples measured after the VST (post-VST, post+15, post+30) was computed. The difference between the peak level and pre-VST sample cortisol level was taken as an indicator of individual cortisol increase. Proportions of responders vs. non-responders were compared. Responders were defined as participants with a minimal cortisol increase of 15.5% from the pre-VST level to the maximal response level (Miller et al., 2013).

#### Gaze behavior.

We used the eye-tracking system implemented within the HMD (VIVE SRanipal SDK, HTC corporation) for measurement of gaze behavior. Areas of interest (AOIs) were predefined and attached to all objects and agents in the virtual room. Gaze behavior was analyzed offline using a custom MATLAB script (v R2022a, The MathWorks, Inc., Natick,MA, USA) which categorized gaze as fixation or saccade behavior. Fixations were defined using both velocity (<75°/s) and gaze duration (>=140 ms) criteria [51]. We computed the latency from speech onset until the first fixation on the currently speaking agents, percentage of (in)correct fixations, as well as the dwell time on speaking agents/ social AOIs. Eye tracking data from two participants had to be excluded due to technical errors.

### Statistical analyses

Statistical analyses were conducted using the R environment [50]. For all hypotheses, first, mixed ANOVAs were computed, and Greenhouse-Geisser correction was applied in cases of violations of sphericity. Then, post-hoc t-tests were computed to follow up on significant effects. Directed one-sided t-tests for independent samples were computed to gain evidence on the superiority of externalized auralizations compared to non-externalized ones (H1, H2, and H3). When the requirements for parametric tests were not fulfilled, the Mann-Whitney-U-Test, as a non-parametric equivalent, was computed and the Wilcox test (W) was reported. Holm procedures were used to correct for multiple comparisons (H2). For all hypothesis tests except for H6, audio manipulation was analyzed with two levels: non-externalized vs. externalized, including data from individual and generic audio groups. Interaction effects of Stress-by-Audio-by-Time on social presence were analyzed for the hypothesis on differential effects of auralizations and stress (H4). For the hypothesis tests concerning a higher stress reaction in the externalization group (H2), we only included data from the high-stress group, and exploratory investigated possible effects for the high- and low-stress groups. For the tests of non-superiority of simulations based on individualized HRIR in comparison to generic HRIR (H5), independent sample t-tests were computed with regard to the above-mentioned outcome variables. If one model resulted in significant differences, the equivalency hypothesis was rejected. Null hypothesis significance testing does not allow a conclusion on equivalence. Therefore, we additionally computed Bayes Factors for independent t-tests to investigate whether the equivalency hypothesis is more probable than the difference hypothesis. The BayesFactor package [52] with the default Cauchy prior distribution was used, and the null-hypothesis was tested against the directed hypothesis of superiority either of externalized auralizations (H1) or individual BRIRs (H5). Bayes Factors greater than three were regarded as confirmatory since indicating moderate evidence [53]. For H6 (differential effects of social anxiety), general linear models on social stress with the predictor of social anxiety were computed for both audio condition groups.

## Results

### Manipulation check

**Fig 3:**
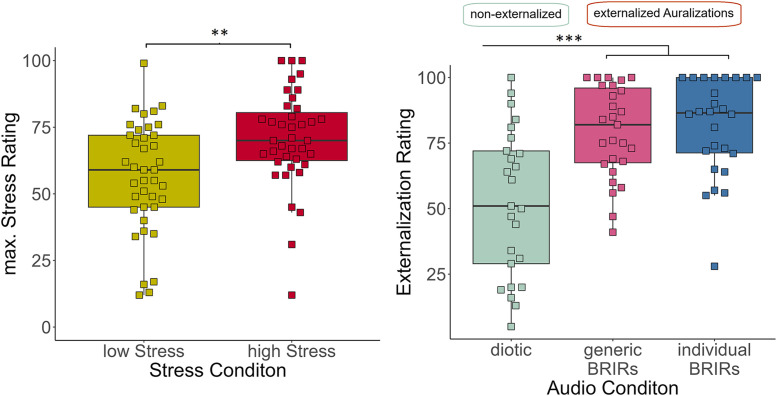
Manipulation check. On the left, maximal stress ratings per stress condition are displayed, on the right, externalization ratings per audio condition.

First, we checked whether our intended manipulations were successful (see Fig 3). Indeed, the high-stress group reported significantly more maximal stress than the low-stress group (W = 450.5, p = .002, d = 0.351, n1 = 39, n2 = 39). Also, the audio manipulation was successful; the externalized auralizations (including individual and generic BRIRs) were rated significantly higher as externalized than the non-externalized one (W = 1044, p < .001, d = 0.464, n1 = 53, n2 = 25).

### Social presence

**Fig 4:**
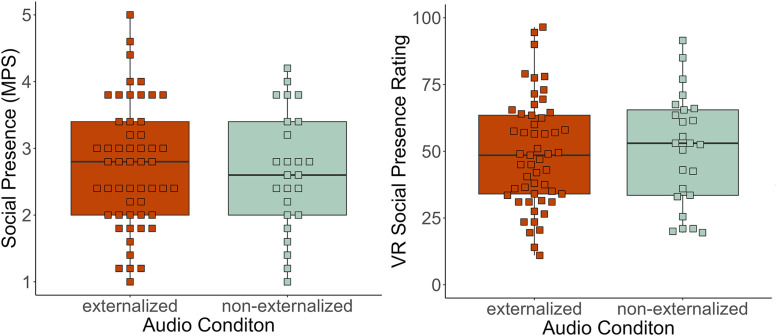
Social Presence. On the left: measured with the subscale of the MPS; on the right: with rating scales within the VR scene.

Neither audio nor stress condition nor their interaction affected social presence measured via the MPS questionnaire conducted after the experiment, see Fig 4, *Audio*: F(1, 74) = 0.11, p = .739, $\eta_{p}^{\text{2}}$ < 0.01; *Stress*: F(1, 74 = 0.00, p = .994), $\eta_{p}^{\text{2}}$ < 0.01; *Audio x Stress*: F(1, 74) = 0.15, p = .702, $\eta_{p}^{\text{2}}$ < 0.01. Social presence was not significantly higher in participants listening to externalized auralizations (M = 2.73, SD = 0.93) compared to those listening to non-externalized auralizations (M = 2.66, SD = 0.89), t(48.65) = −0.35, p = .365, d = 0.08. In addition to the MPS questionnaire, social presence was assessed with a single-item rating in VR directly after an interaction (two times). Again, neither a significant main effect of *Audio* was found, F(1, 74) = 0.14, p = .709, $\eta_{p}^{\text{2}}$ < 0.1; nor of *Stress*, F(1, 74) = 2.81, p = .098, $\eta_{p}^{\text{2}}$ = 0.04; nor a significant interaction between *Audio* and *Stress*, F(1, 74) = 0.56, p = .458, $\eta_{p}^{\text{2}}$ < 0.1. The mean social presence rating during VR was not higher in the externalized audio group (M = 49.1, SD = 20.4) than in the non-externalized audio group (M = 50.8, SD = 20.8), t(46.32) = −0.32, p = .373, d = −0.08. To sum up, we could neither confirm the hypothesized superiority of externalized auralizations concerning social presence, nor any differential effects of Audio and Stress on social presence.

We additionally computed Bayes factors (BF) for independent t-tests to gain further insights on the (null-)effects of auralizations in our VST. For social presence measured via the MPS, a BF = 3.82 for equivalency of audio conditions was found, and for social presence measured via VR rating, a BF = 3.10 was found. Therefore, moderate evidence was gained that both audio conditions evoked equivalent levels of social presence.

**Fig 5:**
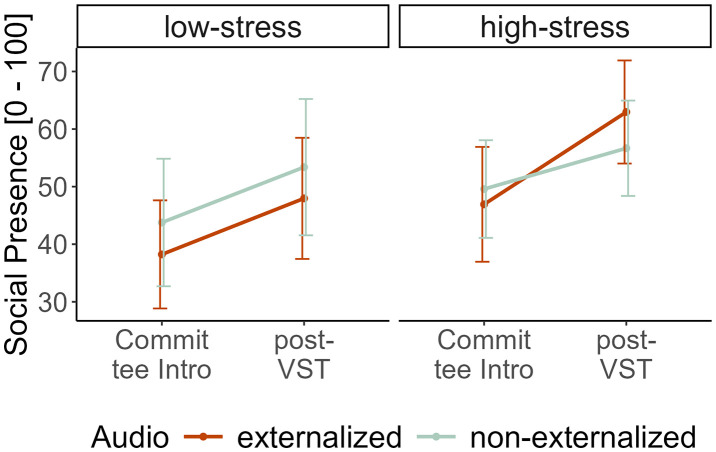
Social Presence rating as a function of stress and audio manipulation at two different measurement time points. Error bars indicate the standard error.

Furthermore, a repeated-measures ANOVA was computed, including the different time points of social presence ratings to gain insights into the time course of social presence and a possible interaction with *Stress*. A significant main effect of *Time,* F(1,148) = 7.90, p = .006, $\eta_{p}^{\text{2}}$ = 0.05; and *Stress,* F(1,148) = 4.71, p = .032, $\eta_{p}^{\text{2}}$ = 0.03, was found. Fig 5 indicates that social presence increases throughout the VST and is higher in the high-stress group.

We conducted exploratory analyses on additional indicators of the quality of experience in VR, including physical presence, perceived realism, and subjective audio quality. Detailed results of these analyses are provided in the supporting information (S1 File). Similar to social presence, physical presence also showed time-related effects, with a general increase observed over time (see S1 Fig). Presence (social and physical) positively correlated with acoustic realism and acoustic presence. Interestingly, acoustic realism and tone richness, but not acoustic presence, were positively affected by externalized auralizations. Finally, audio liking and speech intelligibility were affected by externalization and stress, with these two conditions interacting marginally significantly (see S2 Fig). Furthermore, time-lagged Pearson’s correlations across five measurement points between stress and social or physical presence were computed to investigate their causal relationship, see S3 Fig Numerically, the strongest relationship between both social and physical presence and stress was found between the rating after the committee introduction and the subsequent stress rating (post VST). This indicates that the more participants experienced the initial virtual social interaction as if really being in front of a job interview committee, the more stress they experienced later during the VST.

### Stress induction

#### Salivary cortisol levels.

**Fig 6:**
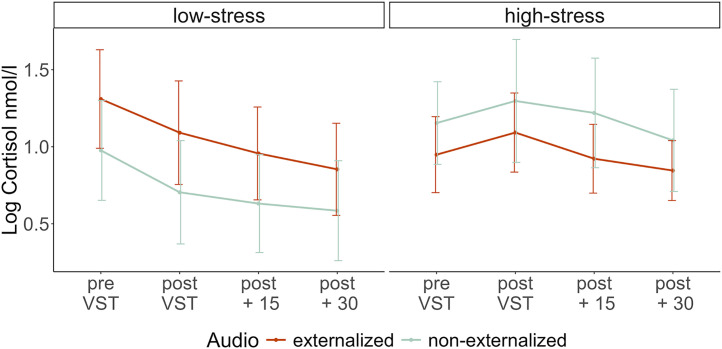
Log-transformed mean salivary cortisol (nmol/l) in response to the VST as a function of audio and stress. Error bars indicate the standard error.

A repeated measures ANOVA was conducted to examine the effect of *Time* (of salivary sample), *Stress* (high vs. low), *Audio* (externalized vs. non-externalized), and their interactions on the salivary cortisol level, while controlling for sex, hormonal contraception, and age. We found a significant interaction effect of *Time* and *Stress*, F(1, 73)=7.49, *p* = .008, $\eta_{p}^{\text{2}}$ = 0.09, confirming that the cortisol increase specifically occurred in the high-stress condition (see Fig 6). An effect size of d = 0.41 of VST (pre vs. peak) on salivary cortisol was found in the high-stress group. While sex also significantly influenced the salivary cortisol level, F(1, 70) = 6.94, p = .010, $\eta_{p}^{\text{2}}$ = 0.09, neither *Audio*, F(1, 70) = 0.27, p = .605, $\eta_{p}^{\text{2}}$ < 0.01, nor *Time*, F(1, 70) = 0.03, p = .864, $\eta_{p}^{\text{2}}$ < 0.01; nor any of the other covariates, *HC*: F(1, 70) = 0.64, p = .437, $\eta_{p}^{\text{2}}$ < 0.01, *Age*: F(1, 70) = 0.640, p = .167, $\eta_{p}^{\text{2}}$ = 0.03, had a significant main effect on salivary cortisol level. Also, the interaction effect between *Audio* and *Stress* did not reach significance, F(1, 73) = 3.23, p = .077, $\eta_{p}^{\text{2}}$ = 0.04. Furthermore, neither the interaction between *Audio* x *Time* was significant, F(1, 73) = 0.05, p = .828,$\eta_{p}^{\text{2}}$ < 0.01; nor the three-way interaction between *Audio* x *Time* x *Stress,* F(1, 73) = 0.05, p = .826, $\eta_{p}^{\text{2}}$ < 0.01. Contrasting our hypotheses, the increase in salivary cortisol level was not significantly higher in the externalization audio group (M = 0.17, SD = 0.68) than in the non-externalization group (M = 0.17, SD = 0.42), t(37) = −0.02, p = .507.

Additionally, we analyzed whether the responder rates differed between experimental groups (see Table 2). A binary logistic regression model revealed that participants in the high-stress condition had a significantly higher probability of being classified as responders compared to the low-stress condition, *b* = 1.09, *SE* = 0.54, *z* = 2.02, *p* = .043, *OR* = 2.99. Neither an effect of *Audio* nor an interaction between *Audio* and *Stress* was found. See S4 Fig for the cortisol response in responders only.

#### Heart rate.

**Fig 7:**
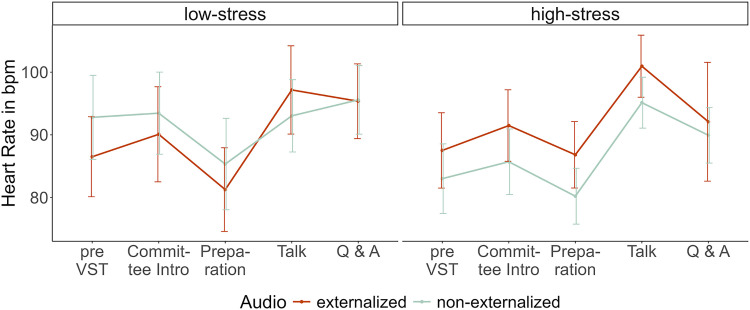
Mean heart rate in beats per minute in response to the VST as a function of stress and audio manipulation. Error bars indicate the standard error.

A repeated-measures ANOVA including the measurements from all five time points revealed a significant interaction between *Time* and *Stress*, F(4, 256) = 2.64, p = .035, $\eta_{p}^{\text{2}}$ = 0.04, as well as a significant main effect of *Time*, F(4, 256) = 31.61, *p* < .001, $\eta_{p}^{\text{2}}$ = 0.33. When only including two measurements (as preregistered), the baseline and the individual peak heart rate after start of the VST, the model resulted in different significant effects. While *Time* also significantly affected heart rate, F(1, 64) = 139.35, p < .001, $\eta_{p}^{\text{2}}$ = 0.69; this was not found for the interaction between *Time* and *Stress*, F(1, 64) = 0.16, p = .692, $\eta_{p}^{\text{2}}$ < 0.01. Neither *Stress*, F(1, 64) = 0.37, p = .547, $\eta_{p}^{\text{2}}$ < 0.01; nor *Audio*, F(1, 64) = 0.17, p = .679, $\eta_{p}^{\text{2}}$ < 0.01; nor *Audio* x *Stress*, F(1, 64) = 1.17, p = .283, $\eta_{p}^{\text{2}}$ < 0.01; nor *Audio* x *Time*, F(1, 64) = 3.778, p = .056, $\eta_{p}^{\text{2}}$ = 0.06; nor *Audio* x *Time* x *Stress*, F(1, 64) = 1.98, p = .164, $\eta_{p}^{\text{2}}$ = 0.03; significantly affected heart rate. As illustrated in Fig 7, the difference between the two models might mainly be due to the preparation phase. During the preparation phase, the heart rate seems to decrease only in the low-stress group (sitting down and reading the answers) while remaining relatively constant in the high-stress group (also sitting down but preparing the talk). This may reflect differences in cognitive demand during the preparation [54]. Again, contrary to our hypotheses, the increase in heart rate was not significantly higher in the externalized audio group (M = 14.8, SD = 11.5) than in the non-externalization group (M = 13.7, SD = 6.8, t[31] = 0.28, p = .392).

#### Subjective stress.

**Fig 8:**
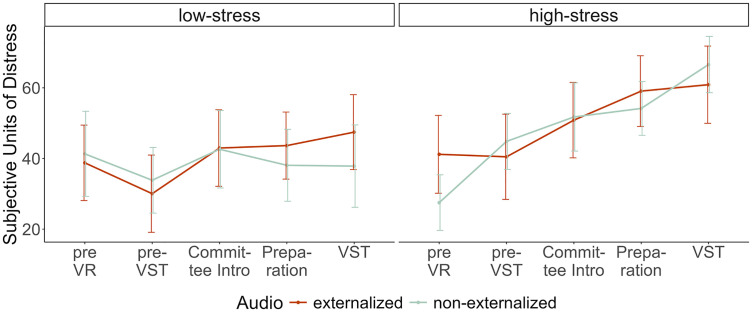
Mean stress rating as a function of stress and audio manipulation at five different measurement time points. Error bars indicate the standard error.

A repeated-measures ANOVA including all five measurement times of stress ratings revealed a significant main effect of *Stress*, F(1, 74) = 5.90, p = .018, $\eta_{p}^{\text{2}}$ = 0.07; and *Time*, F(4, 296) = 15.65, p < .001, $\eta_{p}^{\text{2}}$ =.17. Also, a significant interaction between *Time* and *Stress* was found, F(4, 296) = 4.91, p < .001, $\eta_{p}^{\text{2}}$ = 0.06. As can be seen in Fig 8, subjective stress levels are higher and increase more continuously in the high-stress group.

As preregistered, only the baseline and the individual peak after stress instruction rating were included to test the hypothesis of a stronger subjective stress reaction in the externalized auralizations condition. This model again revealed a significant effect of *Time*, F(1, 74) = 99.34, p < .001, $\eta_{p}^{\text{2}}$ = 0.57, a significant interaction between *Time* x *Stress* F(1, 74) = 11.19, p = .001, $\eta_{p}^{\text{2}}$ = 0.13, and of *Time* x *Stress* x *Audio*, F(1, 74) = 5.05, p = .028, $\eta_{p}^{\text{2}}$ = 0.06. Neither a main effect of *Stress*, F(1, 74) = 1.80, p = .184, $\eta_{p}^{\text{2}}$ = 0.02; nor of *Audio*, F(1, 74) = 1.48, p = .227, $\eta_{p}^{\text{2}}$ = 0.02; nor an interaction between *Audio* x *Time*, F(1, 74) = 0.01, p = .938, $\eta_{p}^{\text{2}}$ < 0.01; nor between *Audio* x *Stress*, F(1, 74) = 0.20, p = .658, $\eta_{p}^{\text{2}}$ < 0.01; was found. Again, contrasting our hypotheses, the increase in subjective stress was not higher in the externalized auralizations group (M = 29.8, SD = 23.9) than in the non-externalized group (M = 42.3, SD = 13.3, t[37] = −1.69, p = .951, −0.59.

### Gaze behavior

Only data from the high-stress group was analyzed due to the methodological differences (reading of preformulated answers in the low-stress group). Eye-tracking data from the question and answer phase were analyzed since offering 30 similar trials. In the externalized audio group, the mean latency of the first fixation (in ms) on the speaking agent was not significantly shorter (M = 1127, SD = 213) than in the non-externalized audio group (M = 1078, SD = 193); t(20) = 0.69, p = .752, d = 0.24.

Exploratorily, possible differences in the number of fixations on correctly identified speaking agents were investigated. Again, no significant difference was found between the externalized audio group (M = 25.6, SD = 3.7) and the non-externalized audio group (M = 24.2, SD = 8.0); $X^{\text{2}}$(11) = 16.14, p = .136.

### Social anxiety

As preregistered, we analyzed whether the regressional weight of participants’ social anxiety on social stress is differentially dependent on the externalized auralizations (and the stress manipulation), using three general linear models. Social anxiety, indexed by the continuous SPIN total score, was not a significant predictor of the increase in salivary cortisol, heart rate, or subjective stress from baseline to post or during VST measurement, nor was the interaction with audio or stress.

**Fig 9:**
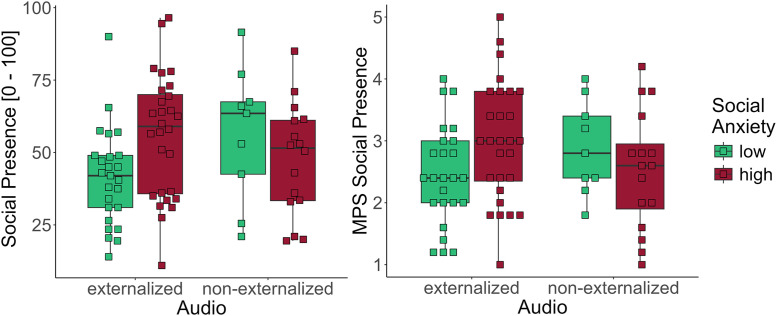
Social Anxiety and Audio. Social presence rating (on the left) and subscale of the MPS (right) as a function of audio and social anxiety (SPIN median split).

Furthermore, we exploratorily analyzed whether social presence, gaze behavior, or stress response varied as a function of social anxiety and audio condition. For this purpose, a median split was conducted to classify participants as lower or higher socially anxious. Detailed results are provided in the supporting information. Briefly, for social presence, an interaction between social anxiety and auralizations was found, with higher socially anxious participants reporting higher social presence, but only when externalized auralizations were used, see Fig 9. Moreover, higher socially anxious participants showed shorter latencies from speech onset until first fixation on the speaker, possibly reflecting hypervigilance [55]. Concerning stress indicators, a main effect of social anxiety (low vs. high) was found for heart rate and subjective stress, but no interaction effect between social anxiety and time or audio.

### Equivalency of externalized auralizations

We expected non-superiority of auralizations based on individual HRIRs in comparison to generic HRIRs concerning all outcome variables. In addition to independent sample t-tests, we computed Bayes Factors on the probability of the equivalency hypothesis. These results were presented at the 51^st^ German Acoustical Society meeting (DAGA) and published (non-peer-reviewed) within the conference proceedings [56]. As can be seen in Table 3, the Bayes Factors for H0 for all outcome variables are larger than 1, implicating at least anecdotal evidence in favor of equality versus a difference [53]. However, the critical threshold of 3 was only reached for social presence and heart rate.

**Table 3:** Means of outcome variables in individual and generic HRIRs audio conditions and the Bayes factor for an independent samples t-test of H0.

	Individual HRIRs (n = 26)	Generic HRIRs (*n* = 27)	Bayes Factor H0
*Social presence (MPS)*	*2.62*	2.84	6.21
*Stress Response – Increase of*			
*Salivary cortisol*	0.44	−0.26	1.73
*Heart rate*	13.22	17.78	4.53
*Subjective stress*	29.15	21.67	1.16
*Visual spatial attention*	1128	1073	1.97

## Discussion

### Summary

We investigated the effects of binaural auralizations which are perceived as externalized on presence and stress reactions in a virtual stress scenario. Participants completed a virtual job interview, which was either intended to induce high or low social stress, while the auditory scene was manipulated as externalized and realistic or as a non-externalized control condition. As intended, all three indicators for stress, salivary cortisol, heart rate, and ratings, reflected a response to the VST. Also, as expected binaural auralizations were perceived as more externalized and with higher acoustic realism compared to the diotic control condition. However, there was no effect of audio condition on social presence and neither on measures of stress response such as salivary cortisol, heart rate, subjective stress ratings, and visual spatial attention. Social presence was higher in the high-stress group and increased during the VST. Also, physical presence was found to increase with time spent in VR, or alternatively, as the VST progressed. Although neither social presence nor presence ratings were affected by the audio conditions, an interaction effect between audio and the participants’ level of social anxiety level was found on an exploratory basis. Social presence was increased in participants with high social anxiety, but only when externalized auralizations were used. Also, the latencies of first fixations on agents after their speech onset were lower for higher socially anxious participants. Social anxiety (high vs. low) had a main effect on heart rate and stress ratings, but not on salivary cortisol. We also exploratorily evaluated the subjective quality of the acoustic scene and the VR experience via ratings (see S2 Table). Interestingly, acoustic realism was positively correlated with both physical and social presence. A similar pattern was observed for acoustic presence. Furthermore, high stress decreased speech intelligibility and audio liking compared to low stress, while externalization increased speech intelligibility and audio liking compared to non-externalized sound. Last, evidence was gained that individualization of binaural auralizations is not superior to the use of generic binaural auralizations concerning all measured variables. To sum up, the present study does not support the claim that externalized binaural auralizations increase stress responses and social presence in a stressful virtual interaction. Instead, we identified specific relations between binaural auralizations and quality of experience in VR, as well as between interindividual differences related to social anxiety and stress responses.

### Effects of binaural auralizations on virtual interactions

In contrast to our hypotheses, social presence did not differ between externalized and non-externalized auralizations. As outlined in the beginning, we expected increased social presence due to increased social realism when speech is perceived at the position where agents are located. Although the audio manipulation was effective, with binaural auralizations perceived as externalized and the respective virtual scene as more realistic, it had no measurable impact on presence. In a review, a positive effect of audio quality on social presence was summarized [10]. Non-VR applications such as a first-person shooter video game [57] and business-teleconferences [58] were investigated. In complex audiovisual scenes, the impact of externalized auralizations may be more limited. This may be particularly true for high-arousal scenarios [27] like the VST. Indeed, in the current study, not only the high-stress group but also the low-stress group experienced increases in subjective distress (on average by 30%), which was also found to affect social presence [27]. Differences in immersion were found to have larger effects on presence in non-emotion VR scenarios [26]. Possibly, (between-subject) differences in the acoustic scene may not be salient enough when embedded in a photorealistic visual scene in which demanding tasks must be accomplished. Furthermore, sound externalization was not task relevant. Under increased arousal, participants may have allocated their limited cognitive resources to threat-relevant stimuli, potentially the committee’s neutral, feedback-free behavior. Attention is probably shifted towards nonverbal social rather than spatial cues. Notably, the TSST has proven effective even in teleconference formats without spatial co-presence of the committee, highlighting the task-irrelevance of spatial audio for eliciting social threat. [59]. Regarding the impact of binaural auralizations on social presence in complex audiovisual environments, findings are inconsistent. While one study found increased social presence with externalized auralizations in a virtual seminar room where participants had to localize the speaker [3]; another VR study involving dyadic problem-solving found no such effect, even when participants were encouraged to move around in order to experience spatial audio [11]. In the former, externalization was task-relevant; in the latter, it was not.

Beyond the elevated arousal in our study and the task-irrelevancy of spatial audio, conceptual aspects of presence should be addressed to clarify the role of externalized auralizations in VR. The association between acoustic realism and physical presence was stronger than with social presence. This suggests that participants linked realistic sound more to the overall VR environment than to the virtual agents. The more the speech was perceived as occurring in a real room, the stronger the reported sense of ‘being there.’ Thus, spatial audio may influence physical presence more than social presence. While the MPS social presence subscale was found to be sensitive to (large) social realism manipulations in a previous study, specificity to physical presence manipulations was low [60]. This indicates that future studies should investigate the effects of spatial audio on experience in VR in a broader sense and concerning spatial presence.

Overall, moderate levels of social presence were found, with average ratings near the scale midpoint. While consistent with previous VR studies [60,61], this suggests that about half the participants lacked a clear sense of co-presence, indicating room for improving virtual social interactions. Alternatively, such levels may be expected when no deceptive instruction suggests interaction with a real human. Social presence encompasses an increasingly broad range of phenomena [62], and without deception, low ratings may reflect perceived non-human actorhood [62] regardless of audio realism. Future studies should complement the MPS with more specific items on salience, social realism, and involvement to better assess implementing binaural auralizations in virtual social interactions. Although integrating AI to simulate artificial humans may enhance interaction [63], it requires careful monitoring through refined social presence measures.

Next, we expected the VST to evoke stronger emotional responses due to increased social presence when immersivity is higher (by implementing externalized auralizations) as suggested by previous work [2,10,22,64]. However, it was also found that not the presence of others, but rather the evaluative component of social presence, determined the response to stressors [65]. As no audio effects were found on social or physical presence, nor visual attention, the absence of an effect on stress aligns with these findings.

### VST paradigm

The present study presents a modified version of the TSST which is adapted for the manipulation of audiovisual VR. Our findings demonstrate a robust stress reaction in this paradigm which was observed on subjective, physiological, and neuroendocrine measures. Concerning subjective stress, as expected, the high stress group showed a higher increase than the low stress group, but unexpectedly, stress also increased in the low-stress group by up to 30% on average. Qualitative reports suggest that the reading-aloud task in front of the committee also triggered social evaluative threat in some of the low-stress participants. Furthermore, the context of a job interview could have been generally perceived as stress-related. In contrast to subjective stress, cortisol stress increased in response to the VST selectively in the high-stress group, but decreased in the low-stress group. Neuroendocrinological measures may capture acute social stress and evaluative threat more distinctly and specifically than ratings.

In line with the literature the current VST resulted in overall cortisol responder rates of 41%; confer rates of 57% in a neuroimaging version of the TSST [66] and 33% to 86% for virtual and in-vivo TSST [13]. Similarly, the absolute cortisol increase (effect size of d = 0.41 in the high-stress group) aligns with prior findings; a meta-analysis [67] reported average VR stress reactivity of d = 0.65 (range: 0.21–1.65).

Cortisol reactivity appears to be modulated not only by the virtual nature of the TSST but also by demographic variables, with greater responses typically observed in males and individuals under 25 [33]. In the present study, the predominance of female participants, despite hormonal control, may have resulted in reduced cortisol responsivity. On the other hand, the young age of the current participants and the high immersivity of the VST could have counteracted this effect [33].

The present paradigm adapted the TSST to increase audio-visual components, and this resulted in deviations from the procedure in the traditional paradigm. The present VST lasted longer (6 min talk, 15 min Q&A) than the traditional TSST (5 min talk, 3 min arithmetic). However, we sampled salivary cortisol about 25 minutes (+ 40, + 55) after the onset of acute stress, which is within the best sample period (30–45 min), with peak responses occurring on average 38 min after TSST onset [33]. Furthermore, the TSST seems to be fairly robust to variations in the length of periods [33]. All in all, we provided a modified version of the VR-TSST, where stress induction is comparable to previous work and where a higher proportion of speech was held by virtual agents, allowing to investigate audio manipulations.

Furthermore, by implementing binaural auralizations, we provided a virtual acoustic environment which was superior to a non-externalized acoustic scene regarding all subjective audio quality features (externalization, liking, intelligibility, acoustic realism, acoustic presence, tone richness). Also, a naturalistic and realistic virtual scene was provided, which was confirmed by qualitative assessments and the VR ratings. Therefore, the current VST can be seen as a helpful tool for investigating the effects of audio (e.g., speech manipulations, synthetic voices, spatialization) on stressful virtual interactions. However, the current findings suggest sound externalization has no substantial effect on stress response.

### Stress and presence

While no main effect of the stress manipulation was found on the mean score of social presence, the effects become significant when the time point of measurement is taken into account. Social presence was higher in the high stress group and increased in both groups during the VST. As mentioned above, the low-stress group also reported increased subjective stress from pre- to post-VST. Also, physical presence increased throughout the VST. Therefore, the increase of presence over time may be due to increasing arousal, which was found to mediate presence [27]. It is not only stated that presence is the basis on which a VR scene results in “real” emotions [28], but also the other way round was found. When arousal is induced, e.g., by displaying a phobia-relevant stimulus, presence in turn increases. The stronger participants’ actual emotional experience is in VR, the more presence they report. Indeed, our supplementary time-lagged correlation analyses indicate effects in both directions. An alternative explanation would be that the more time is spent in VR, the higher the feeling of being there and the feeling of being with others. Since the experimental manipulation of stress level affected (social) presence in the expected direction, arousal and stress are suggested as mediators for higher presence.

### Social anxiety

This implies that participants who react more adversely towards socially stressful situations, meaning participants with high levels of social anxiety, also report more (social) presence. Nonetheless, we found no consistent relationship between social anxiety and social presence. On an exploratory basis, social presence was higher in high socially anxious participants but only in the externalized auralizations condition. These results should be interpreted cautiously, but may indicate a need for investigating differential effects of audio externalization depending on traits.

Concerning stress indicators, heart rate and subjective stress were influenced by social anxiety, whereas cortisol was not. This again implies that these response domains may differentially reflect specific aspects of stress. Cortisol again seems to be a more specific indicator of the biological stress reaction, whereas heart rate may reflect the stressor itself even more. Subjective stress appears as an adequate and sensitive measure of how individuals experience a (social) situation. Indeed, a blunted cortisol stress reaction was found for patients with social anxiety disorder, while subjective stress reports were increased [68]. This dissociation between subjective and cortisol stress reaction may also manifest in participants with varying levels of sub-clinical social anxiety as investigated in the current study. However, this pattern emerged only in the exploratory analyses and not in the preregistered models. These discrepancies likely stem from methodological differences: the preregistered analyses treated social anxiety as a continuous predictor, whereas the exploratory analysis relied on a median split, which may have distorted effect estimates.

### Limitations and future research

The main goal of the study was to investigate the effects of binaural auralizations on presence and specifically social presence, and subsequently social stress and behavior (gaze) in virtual social interactions that induce social evaluative threat. Hence, a specific focus was set on VR applications in the context of social fear. While on an exploratory basis, acoustic realism was correlated with presence, the experimental manipulation of sound externalization had no effects. This implies that in stressful virtual interactions, the implementation of spatialized sound does not make an important contribution to the effectiveness of the scenario. On the one hand, this finding is surprising since audio quality was found to enhance presence [8,9] and social presence. On the other hand, to the best of our knowledge, this is the first study to examine effects of externalized auralization on stressful virtual social interactions. Furthermore, previous work indicated a limited effect of increased immersivity on presence in virtual scenarios which induce high levels of arousal [26]. A similar relation can now be assumed for social presence. In our stressful VR application, no effect of increased immersivity in terms of more realistic and spatial audio was found. Although our study design included a low-stress control condition, this group similarly reported a 30% increase in stress. The problem of designing an appropriate “placebo TSST”, which includes a comparable task but without social evaluative threat, has been discussed before [69]. Often, the social component is removed by omitting the committee. This was not feasible in the current study, given the research goal of investigating audio effects in different stressful social interactions. Therefore, future studies should investigate the effects of binaural auralizations in socially relevant but less stressful situations and further social contexts. Furthermore, specific items for salience, social realism, and involvement should be used [62], as outlined above. Also, contexts in which a stronger influence of the room acoustics can be assumed should be investigated – e.g., concert halls for musicians with stage fright or auditoria for students with public speaking anxiety.

Since sound was found to attract attention to speakers [70], we also expected differences in visual attention depending on the spatiality of sound. Since only in the externalized audio conditions, the direction of the sound source – and therefore a cue about the speaking agent – can be perceived at the moment of the sound onset, we expected a shorter latency of first fixation on speaking agents for this group, and furthermore, sustained attention. The fact that we did not find these effects could be due to the lip synchronization of the agents. These were located in front of the participants, and all of them were within the field of view. It might be that the visual information was so effective that the additional auditory information was not relevant (e.g., ceiling effect). Future studies should evaluate the effect of externalized auralizations on visual attention in virtual interactions in which the speaker location is not immediately visually apparent, making externalized audio more task-relevant.

## Conclusion

We investigated the effect of sound externalization by implementing binaural auralizations in a stressful virtual social interaction. While the VST was efficient in evoking a stress response and the binaural auralizations were shown to be highly realistic and externalized, no audio effects on social presence, stress induction, and visual spatial attention were found. Exploratorily, acoustic realism correlated with presence, and social anxiety interacted with the effects of externalized sound. Implementing spatial sound may not be needed in VR applications in the context of social fear, but it may enhance the realism and the acoustic quality of the virtual environment. Strong evidence is gained that individualization of binaural auralizations is not needed for virtual social interactions. Overall, only medium levels of social presence indicate a need for improvement of the virtual social interactions and a further systematic investigation on factors determining the feeling of being with another when interacting with artificial humans. Future studies should investigate the effects of binaural auralizations on social presence and behavior in virtual social interactions in which sound spatialization may be more salient, task-relevant and crucial for visual spatial attention, and with social presence measurements tailored for interactions with artificial humans.

## Supporting information

S1 Table Job Interview Questions. (PDF)

S2 Table Rating Items. (PDF)

S1 Fig Physical presence rating as a function of stress and audio manipulation at different measurement time points. Error bars indicate the standard error.
(TIFF)

S2 Fig Subjective audio quality ratings as a function of stress and audio manipulation. Error bars indicate the standard error.
(TIFF)

S3 Fig Correlations between stress and presence (top: social; bottom: physical) within and between different measurement time points, *p < .05, ** p < .01, *** p < .001. (TIF)

S4 Fig Salivary Cortisol levels in nmol/l (log transformed) at four different sampling time points for responders only. (TIFF)

S1 File Supplementary Analyses. (PDF)
