# Supplementary material for: Aurally impressed, yet not more stressed: On the relationship between audiovisual realism, social anxiety, and presence in a virtual social stress scenario
Source: PLoS One. 2026 Mar 23;21(3):e0345565. doi: 10.1371/journal.pone.0345565 (PMC13008069; doi:10.1371/journal.pone.0345565)
Supplement: S1 Table — (PDF) [file pone.0345565.s001.pdf]

S1 Table. Job interview questions

N°	Original (German)	English Translation
**1**	Was sind Ihre Stärken und Schwächen?	What are your strengths and weaknesses?
**2**	Wie gehen Sie damit um, wenn Sie einen Fehler gemacht haben?	How do you handle it when you make a mistake?
**3**	Wo sehen Sie sich in 5 Jahren?	Where do you see yourself in 5 years?
**4**	Was war der größte Misserfolg in Ihrem Leben und wie sind Sie damit umgegangen?	What was the biggest failure in your life and how did you deal with it?
**5**	Welches Verhalten einer anderen Person würde Sie auf 180 bringen?	What behavior from another person would make you really angry?
**6**	Wann haben Sie das letzte Mal eine Regel missachtet – und warum?	When was the last time you broke a rule – and why?
**7**	Was haben Sie letzte Woche gelernt?	What did you learn last week?
**8**	Wie stehen Sie zur Legalisierung von Cannabis?	What is your opinion on the legalization of cannabis?
**9**	Warum sind Sie besser als andere?	Why are you better than others?
**10**	Wie sieht Ihr Traumberuf aus?	What does your dream job look like?
**11**	Was müsste passieren, damit Sie den Schritt zu uns bereuen?	What would have to happen for you to regret joining us?
**12**	Was sollte ich unbedingt über Sie wissen?	What should I absolutely know about you?
**13**	Welche 3 positiven Charaktereigenschaften fehlen Ihnen?	Which 3 positive character traits do you lack?
**14**	Wie würden Sie Ihren Arbeitsstil beschreiben?	How would you describe your working style?
**15**	Was tun Sie, wenn Sie merken, die Tagesaufgaben unmöglich zu schaffen?	What do you do when you realize the day's tasks are impossible to complete?
**16**	Wann und wie haben Sie das letzte Mal einen Kollegen kritisiert?	When and how did you last criticize a colleague?
**17**	Welche Aufgabe war für Sie zu schwer – und wie haben Sie das Problem gelöst?	Which task was too difficult for you – and how did you solve the problem?
**18**	Was sind die zentralen Eigenschaften einer guten Führungskraft?	What are the key traits of a good leader?
**19**	Und was sind die zentralen Eigenschaften einer schlechten Führungskraft?	And what are the key traits of a bad leader?
**20**	Was ist Ihr Vorbild?	Who is your role model?
**21**	Haben Sie heute einen schlechten Tag oder treten Sie immer so auf?	Are you having a bad day today or do you always come across like this?
**22**	Erzählen Sie mir etwas von sich, das nicht im Lebenslauf steht.	Tell me something about yourself that's not on your CV.
**23**	Wie mache ich mich in Ihren Augen als Interviewer?	How am I doing as an interviewer in your eyes?
**24**	Was war Ihr bisher schwächster Teil in diesem Vorstellungsgespräch?	What has been your weakest part in this interview so far?
**25**	Welche Frage möchten Sie nicht gestellt bekommen?	Which question would you prefer not to be asked?
**26**	Was führte bisher zu Problemen im Team?	What has previously led to problems in a team?
**27**	Wie haben Sie sich gefühlt, als Sie für Ihre Arbeit kritisiert worden sind?	How did you feel when your work was criticized?
**28**	Was ist Ihr größter Erfolg, der nichts mit Ihrem Beruf zu tun hat?	What is your greatest success that has nothing to do with your profession?
**29**	Wovor haben Sie Angst?	What are you afraid of?
**30**	Wie schaffen Sie so schnell wie möglich eine Vertrauensbasis in einem neuen Team?	How do you quickly build a foundation of trust in a new team?
