# Supplementary material for: Aurally impressed, yet not more stressed: On the relationship between audiovisual realism, social anxiety, and presence in a virtual social stress scenario
Source: PLoS One. 2026 Mar 23;21(3):e0345565. doi: 10.1371/journal.pone.0345565 (PMC13008069; doi:10.1371/journal.pone.0345565)
Supplement: S2 Table — (PDF) [file pone.0345565.s002.pdf]

S2 Table. Ratings

	Rating Item	Question
**Stress**	Stress	How stressed do you feel at the moment?
	Stress – Committee Introduction	How stressed did you feel in front of the committee?
	Stress – post VST	How stressed did you feel during the job interview?
**VR Experience**	Presence	How much do you currently experience virtual reality as if you were really “there”?
	Social Presence	How much did you just feel like you were with other people (regarding the committee)?
	Realism	How realistic did you find the virtual environment?
	Social Realism	How realistic did you find the virtual agents?
**Subjective Audio Quality**	Externalization	Did you hear the audio in your head or outside in the room?
	Acoustic Presence	Did the virtual committee sound as if people present had spoken to you?
	Acoustic Realism	The sound of the speech was like in a real room.
	Audio Liking	How much did you like the sound experience?
	Speech Intelligibility	How did you find the speech intelligibility? (0: hard – 100: effortless)
	Tone Richness	How did you find the tone richness? (0: low – 100: high)
