# Supplementary figures and images for: Aurally impressed, yet not more stressed: On the relationship between audiovisual realism, social anxiety, and presence in a virtual social stress scenario
Source: PLoS One. 2026 Mar 23;21(3):e0345565. doi: 10.1371/journal.pone.0345565 (PMC13008069; doi:10.1371/journal.pone.0345565)

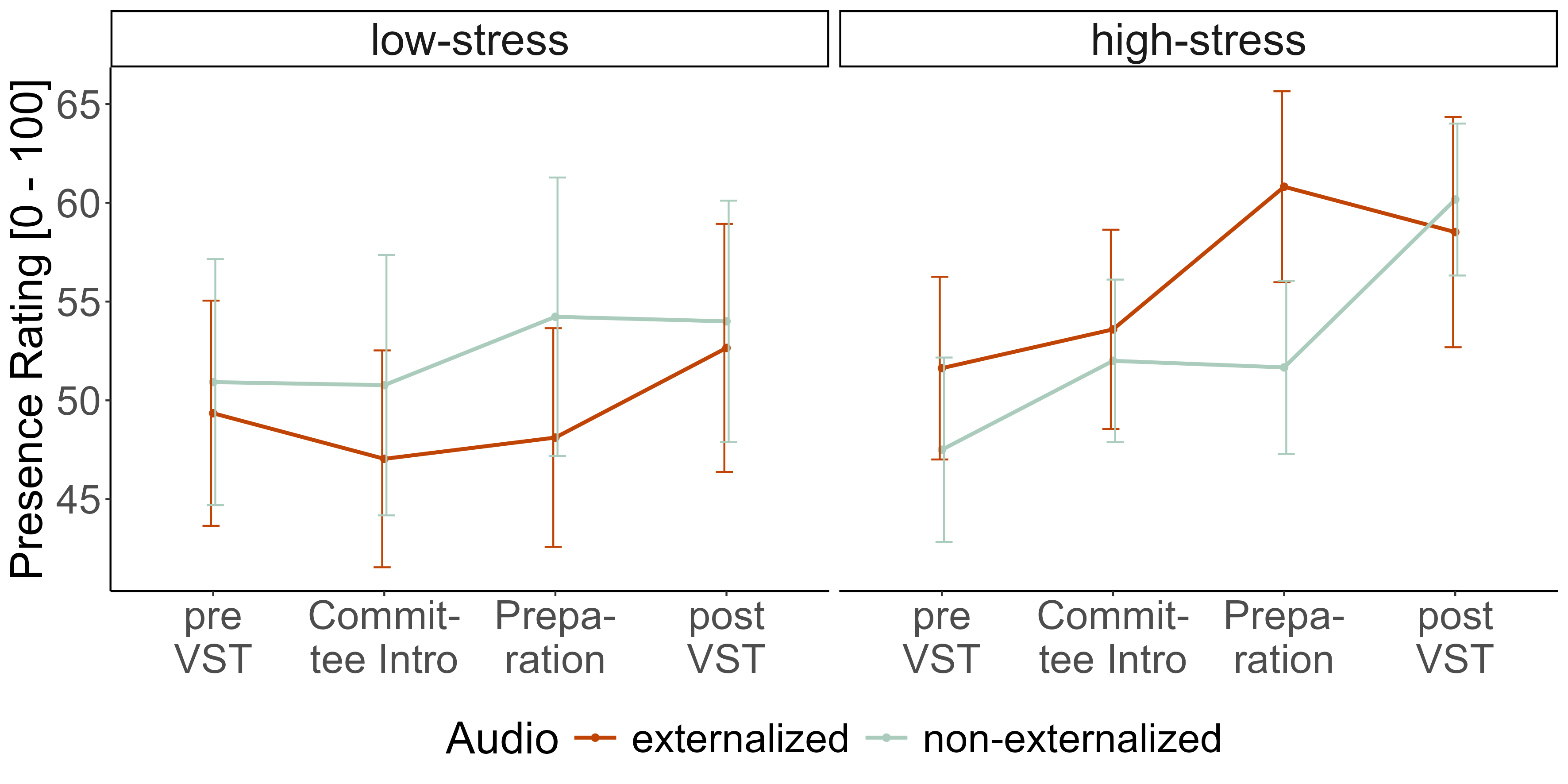

Supplement: S1 Fig — Error bars indicate the standard error. (TIFF) [file pone.0345565.s003.tiff]

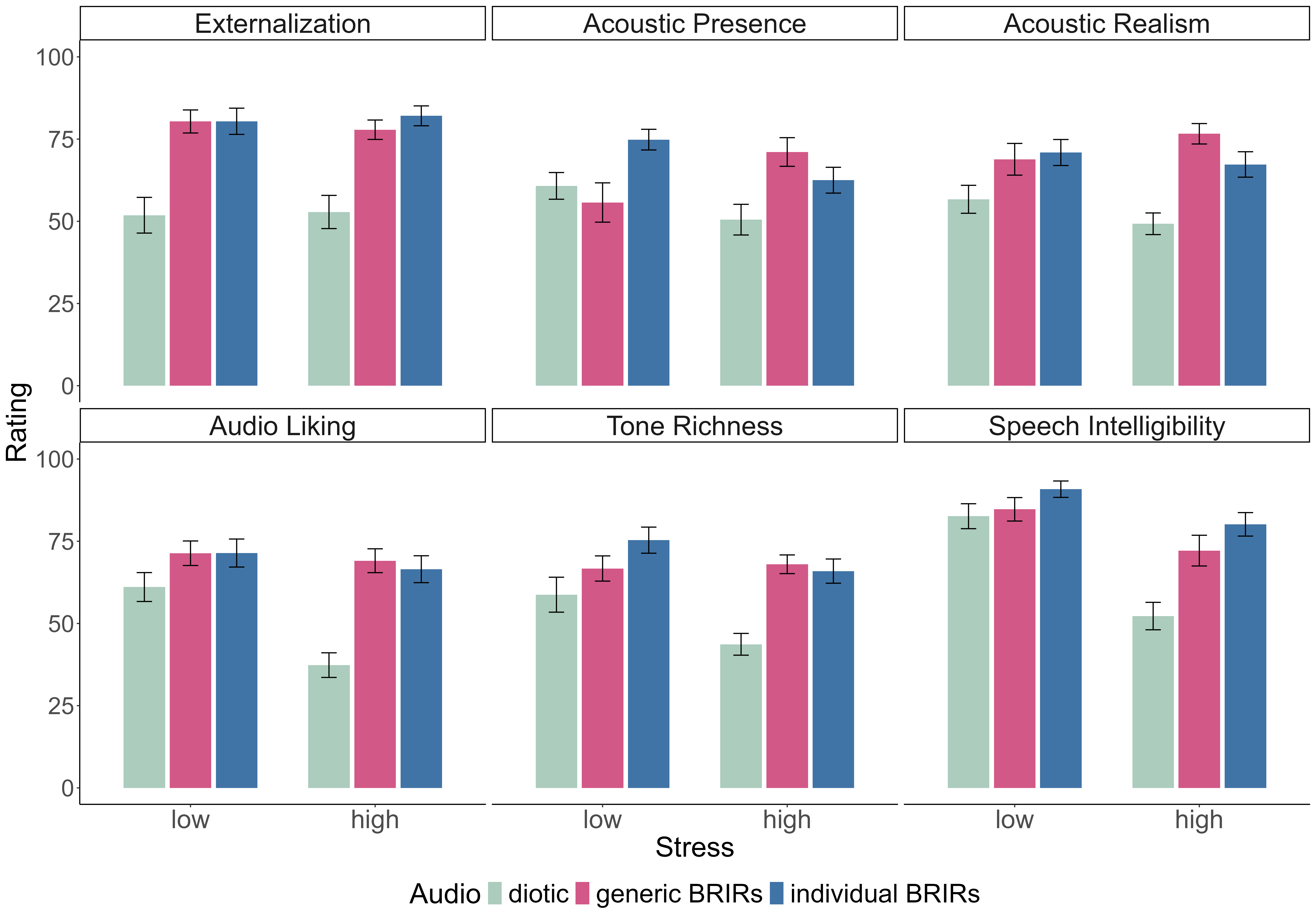

Supplement: S2 Fig — Error bars indicate the standard error. (TIFF) [file pone.0345565.s004.tiff]

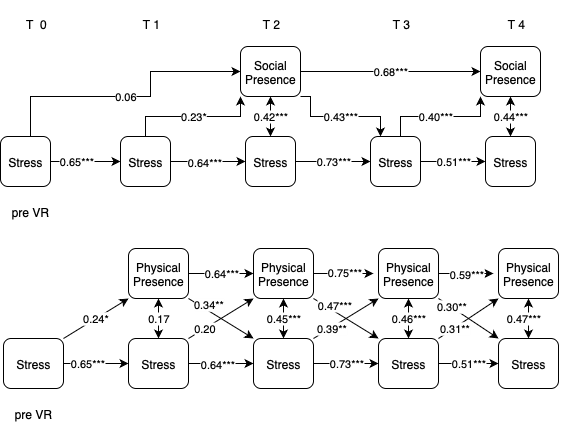

Supplement: S3 Fig — (TIF) [file pone.0345565.s005.tif]

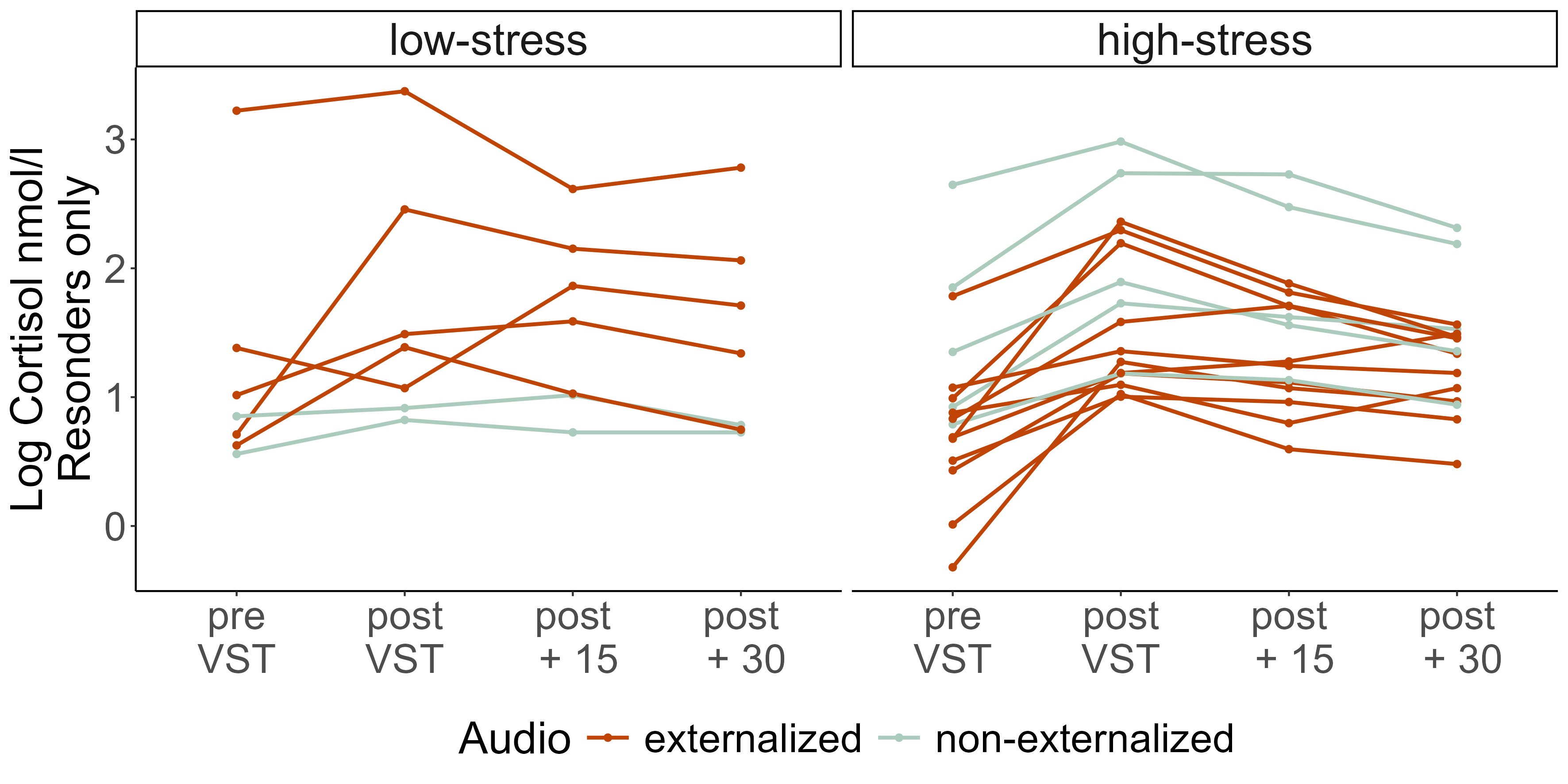

Supplement: S4 Fig — (TIFF) [file pone.0345565.s006.tiff]
