# Supplementary material for: Aurally impressed, yet not more stressed: On the relationship between audiovisual realism, social anxiety, and presence in a virtual social stress scenario
Source: PLoS One. 2026 Mar 23;21(3):e0345565. doi: 10.1371/journal.pone.0345565 (PMC13008069; doi:10.1371/journal.pone.0345565)
Supplement: S1 File — (PDF) [file pone.0345565.s007.pdf]

Supplementary Analyses

Presence, realism, and subjective audio quality

An exploratory mixed ANOVA was computed to investigate the effects of *Audio*, *Time*, and *Stress* on physical presence. A significant main effect of time on physical presence was found, F(2.59, 191.82) = 3.03, p = .038, η_p^2 = 0.05, but no other significant influence.

We exploratorily analyzed the relationship between (social) presence and subjective audio quality. Physical presence measured during the VR scenario positively correlated with acoustic realism at all time points (mean rating) r=0.40, p < .001, and also measured via the subscale of the MPS, r = 0.33, p = .003. While the correlation between acoustic realism and social presence measured with the MPS subscale was not significant (r = 0.21, p = .069), it reached significance for the mean social presence rating during VR, r = 0.23, p = .041. Though both social presence measurements correlated with acoustic presence (MPS, r = .41, p < .001; mean, r = 0.47, p < .001). The externalization rating only correlated with audio liking, r = .48, p < .001, and tone richness, r = 0.34, p = .003. We furthermore conducted mixed ANOVAs to investigate effects of *Stress* x *Audio* on subjective audio quality ratings. A main effect of Audio was found on acoustic realism, F(1, 74) = 11.66, p = .002, η_p^2 = 0.14; audio liking, F(1, 74) = 15.16, p < .001, η_p^2 = 0.17; tone richness, F(1, 71) = 11.24, p = .001, η_p^2 = 0.14; and speech intelligibility, F(1, 74) = 8.38, p = .005, η_p^2 = 0.10. Stress also had a main effect on audio liking, F(1, 74) = 6.86, p = .011, η_p^2 = 0.08; and speech intelligibility, F(1, 74) = 17.28, p < .001, η_p^2 = 0.19. Marginal significant interaction effects of Audio by Stress were found on speech intelligibility (p = .060) and audio liking (p = .058). Last, we examined whether the auralization used had an effect on anticipatory anxiety, which was not the case.

Effects of social anxiety

We explored differential effects of audio externalization and social anxiety on all outcome variables. Therefore, participants were categorized as either low- or high in social anxiety based on their SPIN scores using a median split (median = 28). A mixed ANOVA with the factors *Audio* and *Social Anxiety* (high vs. low social anxiety) revealed an effect of *Audio* by *Social Anxiety* on social presence, both measured with the during-VR rating, F(1, 74) = 5.70, p = .020, η_p^2 = 0.07, and after VR with the subscale of the MPS, F(1, 74) = 4.09, p = .047, η_p^2 = 0.05, see Supplementary Fig 8. Follow-up t-tests revealed significantly higher social presence (MPS) in the high-social anxious (M = 2.99, SD = 0.96) compared to the low-social anxious participants (M = 2.45, SD = 0.81), which was only found in the externalized auralization condition, t(20.23) = -1.05, p = .032. Similar results were found for the mean social presence rating during VR, with the only significant difference found within the externalized auralization. Again, the high-social anxiety group had higher levels of social presence (M = 56.05, SD = 21.1) compared to the low-social anxiety group (M = 41.38, SD = 16.7), t(50.34) = 2.82, p = .007. However, no interaction effects were found for several stress indicators and acoustic quality ratings, all *p*s > .05. Furthermore, we explored the potential influence of social anxiety on stress response. A repeated measures ANOVA with the within-subject factor (time) and the between-subject factor social anxiety (median split via SPIN mean: high vs. low social anxiety) revealed no significant effect on time, stress, or time by stress on salivary cortisol levels. Also, a linear regression model using the raw SPIN value as a predictor was not significant. Concerning heart rate increase, a main effect of social anxiety, F(1, 132) = 8.27, p = .005, η_p^2 = 0.06, and of time, F(1, 132) = 37.58, p < .001, η_p^2 = 0.22 was found, but no interaction effect. Similar effects were found for the dependent variable subjective stress, which was also

significantly affected by time, $F(1, 152) = 20.41$, $p < .001$, $\eta_p^2 = 0.12$, and social anxiety, $F(1, 152) = 56.04$, $p < .001$, $\eta_p^2 = 0.27$, but again no interaction effect was found.

Also, visual attention was affected by social anxiety, but not in interaction with the audio condition. Participants with high social anxiety had a significantly shorter latency (M = 992, SD = 214) until the first fixation on the speaking agents than participants with high social anxiety (M = 1184, SD = 262), $t(63.53) = -3.43$, $p = .001$, $d = 0.73$. Concerning a possible relationship between social anxiety and presence, the SPIN neither correlated significantly with the social presence rating, $r = 0.21$, $p = .063$, nor with the physical or social presence subscale of the MPS, $r = 0.16$, $p = .156$, $r = 0.20$, $p = .084$, respectively. Also, no significant differences between high- and low-social anxious participants concerning presence outcomes were found.
